# The Ha Noi Expert Statement: recognition of maternal mental health in resource-constrained settings is essential for achieving the Millennium Development Goals

**DOI:** 10.1186/1752-4458-5-2

**Published:** 2011-01-07

**Authors:** Jane RW Fisher, Meena Cabral de Mello, Takashi Izutsu, Tuan Tran

**Affiliations:** 1 Deputy Director and Coordinator of International Programs, Centre for Women's Health, Gender and Society, Melbourne School of Population Health, University of Melbourne, Victoria, Australia; 2 Senior Scientist, Department of Child and Adolescent Health and Development, World Health Organization, Geneva, Switzerland; 3 Technical Adviser, United Nations Population Fund, New York, USA; 4 Executive Director, Research and Training Centre for Community Development, Hanoi, Viet Nam

## Abstract

Mental health problems in women during pregnancy and after childbirth and their adverse consequences for child health and development have received sustained detailed attention in high-income countries. In contrast, evidence has only been generated more recently in resource-constrained settings.

In June 2007 the United Nations Population Fund, the World Health Organization, the Key Centre for Women's Health in Society, a WHO Collaborating Centre for Women's Health and the Research and Training Centre for Community Development in Vietnam convened the first international expert meeting on maternal mental health and child health and development in resource-constrained settings. It aimed to appraise the evidence about the nature, prevalence and risks for common perinatal mental disorders in women; the consequences of these for child health and development and ameliorative strategies in these contexts.

The substantial disparity in rates of perinatal mental disorders between women living in high- and low-income settings, suggests social rather than biological determinants. Risks in resource-constrained contexts include: poverty; crowded living situations; limited reproductive autonomy; unintended pregnancy; lack of empathy from the intimate partner; rigid gender stereotypes about responsibility for household work and infant care; family violence; poor physical health and discrimination. Development is adversely affected if infants lack day-to-day interactions with a caregiver who can interpret their cues, and respond effectively. Women with compromised mental health are less able to provide sensitive, responsive infant care. In resource-constrained settings infants whose mothers are depressed are less likely to thrive and to receive optimal care than those whose mothers are well.

The meeting outcome is the Hanoi Expert Statement (Additional file 1). It argues that the Millennium Development Goals to improve maternal health, reduce child mortality, promote gender equality and empower women, achieve universal primary education and eradicate extreme poverty and hunger cannot be attained without a specific focus on women's mental health. It was co-signed by the international expert group; relevant WHO and UNFPA departmental representatives and international authorities. They concur that social rather than medical responses are required. Improvements in maternal mental health require a cross-sectoral response addressing poverty reduction, women's rights, social protection, violence prevention, education and gender in addition to health.

## Introduction

*Safe motherhood is now assured and pregnancy-related deaths are rare in women living in the world's high-income countries. However, most women live in the world's 112 resource-constrained low and lower middle income countries *[1], *where they have less access to family planning services, skilled birth attendants, health care facilities in which to give birth, and basic and emergency obstetric care, than women have in high income countries. They are less likely to have completed primary schooling, to be able to generate an adequate and secure income; and to have had the sexual and reproductive health education that is essential to being able to make autonomous choices about when and how many children they wish to have. They are more likely to live in crowded circumstances and to be poorly nourished and carrying a coincidental burden of infectious diseases including the substantial burden of HIV and AIDS. Their lives are more likely to be constrained by rigid gender stereotypes about appropriate roles and responsibilities for women. Gender-based violence is prevalent in all contexts, but especially in cultures in which girl children and women are devalued and their rights ignored. In these contexts women are at greater risk of dying from pregnancy-related causes, to experience the maternal morbidities of haemorrhage and infection, to give birth to babies who are underweight and not to have access to the health care they need in these circumstances. Their babies are more likely to die and less likely to thrive than babies born in industrialised countries *[2]

The WHO International Classification of Diseases (ICD-10) [3] describes depression as the persistent presence for at least two weeks of a sad lowered mood, loss of interest in activities usually experienced as pleasurable, reduced energy, and at least two other symptoms including: diminished concentration; reduced self-confidence; guilt; a bleak and pessimistic view of the future; ideas or acts of self-harm or suicide; disturbed sleep and diminished appetite. The ICD-10 describes generalized anxiety disorder as characterized by the persistent presence for at least several weeks and usually for several months of apprehension (worries about future misfortune, feeling on edge and having difficulty concentrating); physical tension (restlessness, trembling and inability to relax) and autonomic over activity (lightheadedness, sweating, rapid heart beat, dizziness and a dry mouth).

As the survival of mothers and infants became more certain in high-income settings, awareness of the psychological aspects of pregnancy, childbirth and the postpartum period grew. Mental health problems in women during pregnancy and after childbirth; the links to their sensitivity, responsiveness and caregiving skills and consequences for the health and development of their children have been the focus of substantial research attention in World Bank defined high and upper middle income countries over the past five decades, since being. described first as an 'atypical depression' in the 1960's [4]. It is generally argued that these conditions which are variously described as maternal, antenatal, postnatal (-partum) or perinatal depression and anxiety; or as common non-psychotic perinatal mental disorders are not different from the same conditions occurring at other life phases. However, as observed by Pitt, symptoms of anxiety and irritability are common and the vegetative symptoms of psychomotor retardation and reduced volition and appetite are less common perinatally. Severe occupational fatigue associated with mothering a newborn resembles these mental health problems, compromises functioning and can be mislabeled as depression [5]. About 10% of pregnant women and 13% of mothers-of-infants in high-income countries have significant mental health problems of which depression and anxiety are the most common [6,7]. It was once argued that perinatal mental health problems were not observed in traditional cultures including in low income settings because women were given structured postpartum care which included an honoured status, social seclusion, relief from normal household tasks, gift giving and a mandated period of rest which were protective [8]. Systematic research has only been undertaken more recently in resource-constrained settings and there are major disparities in the volume of local evidence between high income countries almost all of which have substantial high quality data on which to base policy and practice and low income countries, few of which have any data [9]. Nevertheless, the evidence that is available is that common mental health problems are recognizable and, two to three times more prevalent among pregnant women and mothers of infants in resource constrained settings than in high-income countries.

Common mental disorders are multifactorially determined [10] and in general a bio-psycho-social aetiological model is proposed. Individual psychological and biological factors cannot explain wide inter- and intra-country prevalence variations and social factors are the predominant determinants [11]. Perinatal common mental disorders represent substantial social suffering in women. A series of three papers on early childhood development, published in *The Lancet *in January 2007 [12-14], concluded that maternal depression affects substantial numbers of women in resource-constrained countries and that there is consistent epidemiological evidence that in the context of chronic social adversity maternal depression is especially detrimental to child development.

In 2007 the United Nations Population Fund (UNFPA) and the World Health Organization (WHO) collaborated with the Key Centre for Women's Health in Society, a WHO Collaborating Centre for Women's Health in the Western Pacific Region at the University of Melbourne and the Research and Training Centre for Community Development, a national non-government agency in Viet Nam which has maternal mental health as its core priority, to initiate an expert meeting. Its aim was to appraise the evidence about the nature and prevalence of common mental health problems in women in resource-constrained settings, the consequences of compromised maternal mental health for child health and development and of interventions to address these.

## Meeting objectives

The UNFPA-WHO International Meeting on the Interface between Reproductive Health and Mental Health, Maternal Mental Health and Child Health and Development in Resource-Constrained Settings took place in Ha Noi, Viet Nam, between 21 June and 23 June 2007. UNFPA supported the meeting, reflecting its commitment to ensuring that reproductive mental health is included in strategies to address maternal mortality and morbidity; child survival and development, and sexual and reproductive health. Mr Ian Howie, UNFPA Chief of Mission Ha Noi, opened the meeting. Mr Andrew Bruce, Chief of Mission Ha Noi of the International Organization for Migration (IOM), gave an opening address in support of the meeting. Speakers at the opening session included Dr Meena Cabral de Mello, representing the WHO Department of Child and Adolescent Health in Geneva; Dr José Bertolote, representing the WHO Department of Mental Health and Substance Abuse; and Dr Takashi Isutzu, representing UNFPA New York. They emphasized the importance of mental health to endeavors to improve reproductive health, maternal health, and child health and development in resource-constrained settings, and they underscored the value of multiagency, cross-sectoral initiatives.

The meeting brought together the world's leading researchers in this field. They sought to assess the status of knowledge concerning the perinatal mental health problems of women in resource constrained settings, their effects on infants, and the effectiveness of low-cost interventions. The 17 experts came from 11 countries: Australia, Brazil, France, India, Japan, Pakistan, South Africa, Turkey, the United Kingdom, the United States of America, and Viet Nam (see Annex 2 [9]: International and Vietnamese experts). Local participants included 41 policy-makers, researchers, educators, community health development officers and social activists from Vietnamese institutes, governmental agencies, private companies, local nongovernmental organizations and international nongovernmental organizations as well as representatives of United Nations organizations in Viet Nam (see Annex 3 [9]: Vietnamese participants and representatives).

## Meeting outcomes

The first two days of the meeting consisted of four sessions. The international and local experts presented findings from their research in the following areas: (1) prevalence and determinants of maternal mental health problems, (2) consequences of maternal depression for child health and development in resource-constrained settings, (3) interventions to promote maternal mental health in resource-constrained settings and (4) interventions to promote child health and development (see Annex 4 [9]: Papers presented at the meeting and programme agenda).

### 1. Prevalence and determinants of maternal depression

Meeting participants considered the results of a systematic review of the evidence on perinatal mental health in resource-constrained settings. The review was limited to evidence from World Bank-defined low and lower middle income countries, non-psychotic disorders and English-language literature. Almost all the high-income countries of the world have data about the prevalence and determinants of perinatal depression. Such data can serve these countries as the basis for policy and practice. In striking contrast, the review found, only 11 of 112 countries classified as low or lower middle income have any prevalence data.

Systematic reviews of the evidence from industrialized countries have concluded that 10-15% of women experience major depression in pregnancy. The few studies conducted in resource-constrained settings have found rates two to three times higher. Researchers from India, Turkey and Viet Nam presented up-to-date evidence to the meeting. In all three countries at least 25% of mothers of young infants experienced depression or were experiencing clinically significant depressive symptoms.

There are some limitations to the evidence from resource-constrained countries. Many of the studies were undertaken at tertiary-level or university teaching hospitals. Therefore, it might be that only relatively socioeconomically advantaged women were assessed. In some of these countries skilled birth professionals attend relatively few births. No studies, however, involved women who had given birth at home with traditional birth attendants. It is possible that rates of common mental health problems in the general population of mothers of newborns in these contexts are therefore underestimated.

Methodological differences and limitations make comparisons among studies difficult. Some studies have conducted clinical interviews. Other studies have collected data with screening questionnaires. Only some of these questionnaires had been locally validated. Investigations did not all assess the same risk factors. Also, most of the prospective studies assessed mental health in pregnancy only as it constituted a risk for depression after childbirth.

Still, a consistent pattern of high prevalence of common perinatal mental disorders is emerging. Common risk factors include being adolescent, being unmarried, having previous reproductive losses, having an unwelcome pregnancy including pregnancy as a result of forced intercourse, being unable to confide in their intimate partners, lacking emotional and practical support from family members, poverty and lack of personal income generating opportunities, inadequate housing, overcrowding and lack of privacy.

A presentation on the local validation of psychometric instruments in Viet Nam illustrated the need for culturally and psychologically sensitive measures to generate local evidence. Assessment needs to take into account differences in literacy, including emotional literacy, familiarity with the use of self-report instruments, and the establishment of locally appropriate clinical cut-off scores.

The discussion at the end of this session concluded that poor maternal mental health is an especially serious public health concern in resource-constrained settings. This conclusion reverses the once established view that mothers in resource-constrained settings do not experience mental health problems. Participants noted that the common mental health problems of depression and anxiety are predominantly socially determined and that cross-sectoral interventions are therefore needed to address them.

### 2. Consequences of maternal depression for child health and development and the mother-infant relationship in resource-constrained settings

Evidence presented from India, South Africa and the United Kingdom spoke to the impact of maternal postpartum depression on child health and socio-emotional and cognitive development and on the mother-infant relationship. There is consistent evidence from resource-constrained settings that infants of mothers who are depressed are more likely to be of low birth weight, and malnourished and stunted by the age of six months. Studies also report higher rates of diarrheal disease, infectious illness and hospital admission, and reduced rates of completion of recommended immunization schedules in children whose mothers are depressed compared to those whose mothers are not. In combination, these factors are likely to contribute to an increase in child mortality.

There is evidence from developed countries that the mother-infant relationship is compromised when the mother cannot demonstrate warmth and affection, attend to her baby's cues, and respond actively and contingently. In turn, a compromised mother-infant relationship adversely affects the child's cognitive, social, behavioral and emotional development. As yet there is little evidence regarding this linkage from resource-constrained settings.

### 3. Interventions to promote maternal mental health in resource-constrained settings

The psychosocial and physiological demands of pregnancy and caring for an infant make a woman more vulnerable to perinatal mental health problems, especially in adverse circumstances. At the same time, however, routine antenatal and postpartum health services provide an opportunity for heightened and psychologically informed mental health care. Even in the poorest countries there is some provision for antenatal, perinatal, postpartum and infant health care and other primary health care services. Interventions to improve maternal mental health and related child survival, health and development can be integrated into these existing services.

A woman's emotional well-being and social circumstances can be assessed within routine perinatal health care, using either structured questions or appropriately validated and culturally sensitive self-report questionnaires. Stepped intervention protocols, clearly described pathways to care, professional education and health service development are needed. Participants emphasized the importance of an approach that provides care to both mother and baby.

Interventions need to be evidence-based, cost effective, simple and practical and to address both individual needs and family functioning. An intervention being tested in a cluster randomized trial being conducted in a rural area of Pakistan was presented. The intervention involved training village-based community health workers known as Lady Health Workers in a structured cognitive behavioral intervention to treat maternal depression into their routine clinical practice. Evidence from Japan illustrated the importance to maternal and child health of assessing and addressing gender-based violence. Participants also heard how a psychosocial intervention for people who had attempted suicide might be applied to women with perinatal mental health problems. The intervention has been studied in several resource-constrained countries.

Proposed strategies to integrate mental health care into the primary health care system in Viet Nam served as examples. These strategies address research, education and training, policy development and health service development.

The clear evidence that common perinatal mental disorders reflect chronic adversity indicates that social rather than medical responses are required. Improvement in maternal mental health requires a multifaceted social approach and the involvement of multiple sectors including those dealing with development, poverty reduction, human rights, social protection, violence prevention, education, gender, and security, in addition to health. Stepped approaches in which non-pharmacological interventions provided by community-based primary health care workers are the first line response, with clear referral pathways to specialised services for those whose symptoms do not improve.

### 4. Interventions to promote child health and development

To prevent the adverse effects of compromised caregiving on child health and development, interventions must focus specifically on the promotion of infant health and development and strengthening the mother-infant relationship as well as to maternal mental health. Participants considered two such interventions that have been tested in controlled trials in resource-constrained settings. In Khayelitsha, a periurban township in South Africa, trained lay women provided mothers with structured support and education about infant capacities. Mother-infant relationships and child health and development improved. In Porto Alegre, Brazil, a single psycho-educational session about infant behaviour and capacities was conducted individually with mothers of newborns while the mothers were still in hospital. This session improved the mothers' sensitivity and responsiveness to their infants at age six months, compared with mothers who were randomly assigned to a comparison group that received only usual information about infant care.

### 5. Expert statement on maternal mental health and child health and development in resource constrained settings

Overall, the international expert meeting reached a number of conclusions. First, that there is widespread lack of awareness about women's mental health in the perinatal period and its impact on child health and development in resource-constrained settings. Second, that it is essential for each country to have local evidence concerning the nature and prevalence of the problem on which to develop low-cost, non-stigmatizing and accessible interventions. Third, that all resource-constrained countries need cross-sectoral approaches; not only the integration of mental health care into primary perinatal health care, but also strategies to reduce poverty and domestic violence and to promote equality of participation in education and income-generating occupations for women. Fourth, that approaches must be multistranded and include research, education, community-based interventions, health service development, health system strengthening and social policy formation. Finally, that mental health is closely linked to achieving the Millennium Development Goals of improving maternal health, reducing child mortality, promoting gender equality and empowering women, and reducing poverty.

On the final day, in a closed meeting, the core group of 17 international experts and representatives from Viet Nam, UNFPA, WHO and other international agencies drafted The Hanoi Expert Statement entitled *Maternal mental health and child survival, health and development in resource-constrained settings: essential for achieving the Millennium Development Goals *which is reproduced with permission and attached here. It was subsequently reviewed by representatives of relevant departments of UNFPA and WHO and by international authorities in the field. This statement, co-signed by all these stakeholders seeks to inform countries and international agencies about the prevalence and determinants of perinatal mental health problems in women, their consequences for infants, and strategies to address these vital but under-recognized public health problems in resource-constrained settings.

## Competing interests

The authors declare that they have no competing interests.

## Authors' contributions

JF, MCdM, TI and TT convened the meeting on behalf of the four collaborating organizations. They wrote the first draft of the expert statement and, incorporated changes after review by the core group of participants in the meeting, representatives of all relevant departments at the World Health Organization and the United Nations Population Fund, and invited international authorities. All authors read and approved the final manuscript.

## Supplementary Material

Additional file 1 **Maternal mental health and child survival, health and development in resource-constrained settings: essential for achieving the Millennium Development Goals**. This additional file is the Hanoi Expert Statement which is the outcome of the meeting described in this paper. Click here for file
