# Supplementary material for: The Ha Noi Expert Statement: recognition of maternal mental health in resource-constrained settings is essential for achieving the Millennium Development Goals
Source: Int J Ment Health Syst. 2011 Jan 7;5:2. doi: 10.1186/1752-4458-5-2 (PMC3226322; doi:10.1186/1752-4458-5-2)
Supplement: Additional file 1 — Maternal mental health and child survival, health and development in resource-constrained settings: essential for achieving the Millennium Development Goals. This additional file is the Hanoi Expert Statement which is the outcome of the meeting described in this paper. [file 1752-4458-5-2-S1.PDF]

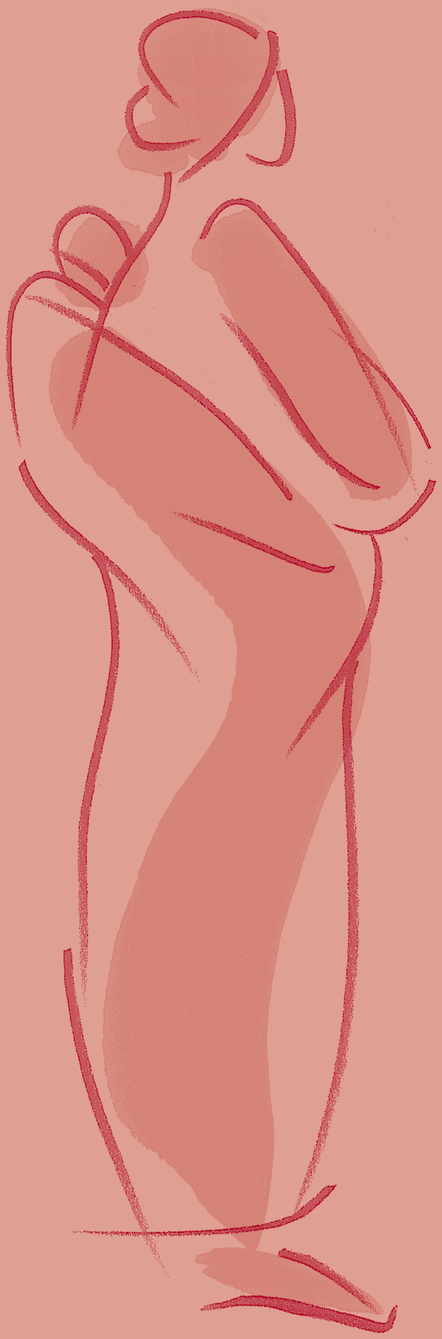

Maternal mental health and child survival, health and development in resource-constrained settings: essential for achieving the Millennium Development Goals

Mental health[1] is fundamental to attaining the Millennium Development Goals of improving maternal health, reducing child mortality, promoting gender equality and empowering women, achieving universal primary education and eradicating extreme poverty and hunger.

Mental health problems are one of the most prevalent and severe, but neglected, complications of pregnancy and childbirth ***(1)***. They make a substantial but currently unrecognised contribution to maternal mortality and morbidity. Suicide is a leading cause of pregnancy-related death in developed countries and of death in young women of reproductive age in some resource-constrained countries ***(2–4)***. One in three to one in five pregnant women and mothers of newborns in developing countries, and about one in ten in developed countries, have significant mental health problems of which depression[2] and anxiety[3] are the most common ***(5–8)***. Moreover, if maternal caretaking capacity is compromised, child survival, health and development are jeopardized ***(9)***. The presumption that culturally-prescribed postpartum care is available and provides mothers of newborns with an honoured status, mandated rest and increased practical assistance, thus protecting mental health ***(10)***, does not reflect reality for many women ***(7)***.

Risk factors for maternal mental health problems

Mental health problems in pregnant women and mothers are predominantly socially determined. The risk factors in developing and developed countries are similar but the prevalence of such factors is higher in the former. Risk factors include:

- poverty and chronic social adversity, including limited education and opportunities for income generation, and crowded living conditions; *(5–7, 11–15*)
- gender-based violence, including emotional, physical and sexual abuse during childhood, family violence, including by intimate partners, and rape; *(5, 16, 17, 18, 19)*
- lack of autonomy to make sexual and reproductive decisions; *(20)*
- unintended pregnancy, especially among adolescent women; *(7, 14, 21)*
- lack of empathy from partners and gendered stereotypes about the division of household work and infant care; *(7, 12, 14, 20, 22, 23)*

World Health Organization

UNFPA

- excessive workloads and severe occupational fatigue; *(24)*
- lack of emotional and practical support or criticism from her own mother or mother in law, or peer group; *(6, 7, 12, 19, 24)*
- gender discrimination and devaluing of women; *(20, 25, 26)* and
- stillbirth, miscarriage and complications of unsafe abortion, pregnancy and childbirth, and persistent poor physical health. *(27)*

Some risk factors appear to be more common in contexts in which there are strong gendered role restrictions on women, including lack of reproductive rights, and giving birth to a daughter in cultures with a preference for sons. *(5, 12, 24, 28)*

Maternal mental health is significantly worse in humanitarian situations or emergencies, especially among refugees and internally displaced people. Sexual and reproductive health is at risk or compromised as a result of deterioration in security and in the functioning of social and health care systems as well as due to a lack of access to appropriate services. Unintended pregnancies increase where access to sexual and reproductive health services is limited. Gender-based violence, including rape, is a common consequence of social unrest and is used as a weapon of war. In addition, people who are trafficked and undocumented migrants are at increased risk of mental health problems and often lack access to health and social services. *(29)*

Impact on mothers and children

Mental health problems constitute a severe burden for both mothers and children. In 2004, perinatal conditions were ranked first, depression fourth and maternal conditions fifth as contributors to the global burden of disease (GBD) experienced by women globally. *(30)* When these co-occur, the human suffering can be extreme. A mother whose mental health is compromised has substantially reduced capacity to care for herself and her infant.

Pregnant women or mothers who have mental health problems often have poor physical health and may have persistent high-risk behaviours including substance abuse. Mothers who are depressed and anxious are less likely to attend for antenatal care or adhere to prescribed health regimens. Although vital registration systems and other systematic data are not available for most developing countries, suicide has been found to make a significant, but under-recognised contribution to pregnancy-related deaths in some of these settings. *(4, 31–34)*

The impact of poor maternal mental health on the developing infant can be severe. Infants are entirely dependent on their caregivers for provision of nutrition, physical care, comfort, social interaction and protection. Infants' neurological, cognitive, emotional and social development are adversely affected if they lack day-to-day interactions with a caregiver who can observe infant cues, interpret these accurately and respond contingently and effectively. *(35–37)* Lifelong capacity to build and maintain satisfactory relationships is established through bonding and attachment in the early years. Without intervention, problematic patterns in relationships can be transmitted and continue across generations. *(38)* Maternal depression in resource-constrained settings is linked directly to lower infant birth weight, higher rates of malnutrition and stunting in six-month-old infants, higher rates of diarrhoeal disease, infectious illness and hospital admission, reduced completion of recommended schedules of immunisation and worse physical, cognitive, social, behavioural and emotional development in children. *(39–45*). In combination these factors contribute to an increase in child mortality.

Mental health and economic development are reciprocally related. Women's mental health is worse if they are not permitted to generate an income, and women with mental health problems can find it difficult to participate economically and socially. This leads to the huge loss of their contributions to society and the economy. There are also clear economic costs to the reduced participation of children who have not been able to reach their full potential. *(46)*

What can be done

Detection, early intervention and treatment strategies are available, but to date have rarely been applied in resource-constrained settings. Even in the least-resourced countries, there is some provision for antenatal, perinatal, postpartum and infant health care and other primary health care services. It is within these existing services that interventions to improve maternal mental health and child survival, health and development can be integrated.

Interventions to improve maternal mental health and promote child health and development include:

- early detection of maternal mental health problems through the use of direct questions about emotional well-being and social circumstances and the use of locally validated screening instruments; *(47)*
- psycho-educational interventions at antenatal and postnatal health care services that combine information provision with psychological support; *(48–50)*

- improvement of the mother-child relationship through enhancement of a mother's sensitivity to infant developmental needs for stimulation, interaction and comfort; *(9, 38, 51, 52)*
- promotion of child health and development through improvements in maternal responsiveness; *(52, 53)*
- improving partner relationships through programmes promoting gender equality and challenging gender-based stereotypes about fathering and household work;
- reducing intimate partner and family violence; *(18)*
- culturally sensitive, solution-focused brief psychological therapies; *(54, 55)*
- improving social support for women through building social networks;
- improving access to education and vocational training for girls and women;
- appropriate treatment of detected depression and anxiety through clearly-defined, stepped protocols that can be managed by primary health care providers; *(56, 57)*
- identification and early referral to specialist services of women at risk of perinatal mental health problems because of a personal or family history of severe mental illness; *(57)* and
- provision of low-cost psychotropic medication to mothers who are extremely depressed and unresponsive to psychosocial interventions, taking into account the risks of these medications to the foetus and to the breastfed infant. *(57)*

Implementation strategies:

- all resource-constrained countries require, as a matter of urgency, local evidence, generated through systematic research and utilizing appropriate methods about the nature, prevalence, social determinants and consequences of maternal mental health problems;
- development and evaluation of improved intervention models which specify the roles and responsibilities of health and non-health sectors;
- health service strengthening, starting with demonstration projects based on the existing evidence;
- capacity development and networking of stakeholders including in the non-health sectors;
- development of a legal and policy framework for the protection of women's mental health;
- stigma reduction and awareness raising among the general population;
- estimation of the financial and human resources to provide these enhanced services on the necessary scale;
- development of indicators to track the progress that countries make in achieving goals to improve maternal mental health;
- support for organizations wanting to implement these recommendations; and
- establishment of adequate funding to support research, implementation and evaluation of community based interventions, mental health education and training for health professionals.

Way forward

Mental health problems are a key determinant of maternal and child mortality and morbidity, but are not currently recognised in existing initiatives to promote maternal health and improve sexual and reproductive health and child health. We believe that the achievement of the Millennium Development Goals to improve maternal health, reduce child mortality, promote gender equality and empower women, achieve universal primary education and eradicate extreme poverty and hunger cannot be achieved unless there is a specific focus on maternal mental health. In doing this it is essential to pay attention to the social determinants of mental health and its key role in maternal health and child survival, health and development, and in increasing the coverage of evidence-based low-cost interventions for maternal mental health problems. Thus, enhancement of maternal mental health requires the involvement of multiple sectors including those dealing with development, poverty reduction, human rights, social protection, education, gender, and security, in addition to health. The *Lancet* has published a recent series of papers about the major global burden of mental health problems in resource-constrained settings. It constitutes an international call to action that there is "No Health without Mental Health".*(26)* Further, mental health is integral to implementing international treaties such as the International Covenant on Economic, Social and Cultural Rights (ICESCR), the Convention on the Rights of Children, the Convention on the Elimination of All Forms of Discrimination Against Women and the Convention on the Rights of Persons with Disabilities, as well as consensus documents such as the Programme of Action of the International Conference on Population and Development (ICPD) and the Beijing Platform for Action of the Fourth World Conference on Women. *(58)*

We call on governments, international organizations and civil societies, informed by WHO's definition of health[4], ICPD's definition of reproductive health[5] and ICESCR's definition of the right to health to take immediate action to address mental health in their endeavours to improve maternal and child health[6], survival and development. Political will, concerted action by global stakeholders and resources are needed now to integrate maternal mental health in strategies to achieve the Millennium Development Goals.

This Statement was developed by the participants in the UNFPA-WHO International Expert Meeting on the Interface between Reproductive Health and Mental Health: Maternal Mental Health and Child Health and Development in Resource-Constrained Settings, held in Hanoi, Viet Nam, 21 to 23 June 2007. The International Expert Meeting was a joint initiative of UNFPA, WHO, the Key Centre for Women's Health in Society, which is a WHO Collaborating Centre for Women's Health in the School of Population Health at the University of Melbourne, Melbourne, Australia and the Research and Training Centre for Community Development in Hanoi, Viet Nam. The International Expert Meeting was convened and the Consensus Statement drafted by Jane Fisher, Key Centre for Women's Health in Society, University of Melbourne, Melbourne, Australia; Meena Cabral de Mello, WHO Department of Child and Adolescent Health and Development, Geneva, Switzerland; Takashi Izutsu, UNFPA, New York, NY, USA and Tran Tuan. Research and Training Centre for Community Development, Hanoi, Viet Nam. The international experts at the meeting and co-signatories to the Consensus Statement were: Abiodun Adewuya, Department of Mental Health, Wesley Guild Hospital, Ilesa, Nigeria; Nazan Aydin, Department of Psychiatry, Ataturk University, Erzurum, Turkey; Bryanne Barnett, Department of Psychiatry, University of New South Wales, Sydney, New South Wales, Australia; José Bertolote, WHO Department of Mental Health and Substance Abuse, Geneva, Switzerland; Peter Cooper, Winnicott Research Unit, University of Reading, Reading, UK; Sudipto Chaterjee, Sangath Centre, Goa, India; Zaeem ul Haq, Johns Hopkins University Center for Communication Programs, Islamabad, Pakistan; Yoshiharu Kim, National Center of Neurology and Psychiatry, Tokyo, Japan; Nguyen thi Nhu Ngoc, Center for Research and Consultancy in Reproductive Health, Ho Chi Minh City, Viet Nam; Doreen Rosenthal, Key Centre for Women's Health in Society, University of Melbourne, Melbourne, Victoria, Australia; Mark Tomlinson, Medical Research Council, Capetown, South Africa; Atsuro Tsutsumi, International Institute of Research and Health, Tokyo, Japan and Jaqueline Wendland, Department of Psychology, University of Paris V, Paris, France. The following international experts reviewed the Consensus Statement and are co-signatories: Patrice Engle, Vikram Patel, Atif Rahman and Tomris Türmen.

Notes:

[1] Mental health is hereby understood as the capacity of individuals to interact with one another, the group and the environment and in ways that promote subjective well-being, the optimal development and use of mental abilities (cognitive, affective and relational) and the achievement of individual and collective goals consistent with justice and the attainment and preservation of conditions of fundamental equality.

[2] The WHO International Classification of Diseases (ICD-10) describes depression as the persistent presence for at least two weeks of a sad lowered mood, loss of interest in activities usually experienced as pleasurable, reduced energy, and at least two of the other common symptoms which include: reduced concentration; reduced self-confidence; ideas of guilt; a bleak and pessimistic view of the future; ideas or acts of self-harm or suicide; disturbed sleep and diminished appetite.

[3] In the WHO International Classification of Diseases (ICD-10) generalized anxiety disorder is characterized by the persistent presence for at least several weeks and usually for several months of apprehension (worries about future misfortune, feeling on edge and having difficulty concentrating); motor tension (restlessness, trembling and inability to relax) and autonomic over activity (lightheadedness, sweating, rapid heart beat, dizziness and a dry mouth).

[4] Health is a state of complete physical, mental and social well-being and not merely the absence of disease or infirmity.

[5] Reproductive health is a state of complete physical, mental and social well-being and not merely the absence of disease or infirmity, in all matters relating to the reproductive system and to its functions and processes.

[6] The right to health is the right of everyone to the enjoyment of the highest attainable standard of physical and mental health.

References

1. Scottish Intercollegiate Guidelines Network. *Postnatal depression and puerperal psychosis. A National Clinical Guideline*. Edinburgh: Royal College of Physicians; 2002.
2. The Department of Health. *Report of the Confidential Enquiries into Maternal Deaths*. London: The Department of Health; 1999.
3. Brockington I. Suicide in women. International Clinical Psychopharmacology 2001;16:S7-S19.
4. Hieu D, Hanenberg R, Vach T, Vinh D, Sokal D. Maternal mortality in Vietnam in 1994-95. *Studies in Family Planning*. 1999;30(4):329-38.
5. Patel V, Rodrigues M, DeSouza N. Gender, poverty, and postnatal depression: a study of mothers in Goa, India. *American Journal of Psychiatry* 2002;159(1):43-47.
6. Rahman A, Iqbal Z, Harrington R. Life events, social support and depression in childbirth: perspectives from a rural community in the developing world. *Psychological Medicine* 2003;33:1161-1167.
7. Fisher J, Morrow M, Ngoc N, Anh L. Prevalence, nature, severity and correlates of postpartum depressive symptoms in Vietnam. *BJOG An International Journal of Obstetrics and Gynaecology* 2004;111:1353-1360.
8. Adewuya A, Afolabi O. The course of anxiety and depressive symptoms in Nigerian postpartum women. *Archives of Women's Mental Health* 2005;8(4):3.
9. Engle P, Black M, Behrman J, Cabral de Mello M, Gertler P, Kapiriri L, et al. Strategies to avoid the loss of developmental potential in more than 200 million children in the developing world. *The Lancet* 2007;369:229-241.
10. Stern G, Kruckman L. Multi-disciplinary perspectives on post-partum depression: an anthropological critique. *Social Science and Medicine* 1983;17:1027-1041.
11. Faisal-Curry A, Tedesco J, Kahhale S, Menezes P, Zugaib M. Postpartum depression: in relation to life events and patterns of coping. *Archives of Women's Mental Health* 2004;7:123-131.
12. Abiodun O. Postnatal depression in primary care populations in Nigeria. *General Hospital Psychiatry* 2006;28:133-136.
13. Edwards G, Shinfuku N, Gittelman M, Ghozali E, Haniman F, Wibisonos S, et al. Postnatal depression in Surabaya, Indonesia. *International Journal of Mental Health* 2006;35(1):62-74.
14. Owoeye A, Aina O, Morakinyo O. Risk factors of postpartum depression and EPDS scores in a group of Nigerian women. *Tropical Doctor* 2006;36:100-103.
15. Fisher J, Tran H, Tran T. Relative socioeconomic advantage and mood during advanced pregnancy in Vietnam. *International Journal of Mental Health Systems* 2007;1.
16. Inandi T, Elcib O, Ozturk A, Egrie M, Polat A, Sahin T. Risk factors for depression in postnatal first year, in eastern Turkey. *International Journal of Epidemiology* 2002;31:1201-1207.
17. Fikree FF, Bhatti LI. Domestic violence and health of Pakistani women. *International Journal of Gynecology and Obstetrics* 1999;65(2):195-201.
18. Garcia-Moreno C, Jansen H, Ellsberg M, Heise L, Watts C. *WHO Multi-Country Study on Women's Health and Domestic Violence Against Women: Initial Results on Prevalence, Health Outcomes and Women's Reponses*. Geneva: World Health Organization; 2005.
19. Hussain N, Beve I, Hussain M, Chaudhry IB, Atif N, Rahman A. Prevalence and social correlates of postnatal depression in a low income country. *Archives of Women's Mental Health* 2006;9:197-202.
20. Rodrigues M, Patel V, Surinder J, de Souza N. Listening to mothers: qualitative studies on motherhood and depression from Goa, India. *Social Science and Medicine* 2003;57:1797-1806.
21. Piyasil V. Anxiety and depression in teenage mothers. *Journal of the Medical Association of Thailand* 1998;81(2):125-129.
22. Aydin N, Inandi T, Karabulut N. Depression and associated factors among women within their first postnatal year in Erzurum province in eastern Turkey. *Women & Health* 2005;41(2):12.
23. Agoub M, Moussaoui D, Battas O. Prevalence of postpartum depression in a Moroccan sample. *Archives of Women's Mental Health* 2005;8:37-43.
24. Chandran M, Prathap T, Muliyil J, Abraham S. Post-partum depression in a cohort of women from a rural area of Tamil Nadu, India. *British Journal of Psychiatry* 2002;181:491-504.
25. Fikree F, Pasha O. Role of gender in health disparity: the South Asian context. British Medical Journal 2004;328:823-826.
26. Prince M, Patel V, Saxena S, Maj M, Maselko J, Phillips M, Rahman, A. No health without mental health. *The Lancet* 2007;370(9590):859-877.
27. Brown S, Lumley J. Physical health problems after childbirth and maternal depression at six to seven months postpartum. *BJOG An International Journal of Obstetrics and Gynaecology* 2000;107:1194-1201.
28. Adewuya A, Fatoye F, Ola B, Ijaodola O, Ibigbami S. Sociodemographic and obstetric risk factors for postpartum depressive symptoms in Nigerian women. *Journal of Psychiatric Practice* 2005;11(5):353-358.
29. Rahman A, Hafeez A. Suicidal feelings run high among mothers in refugee camps: a cross-sectional survey. *Acta Psychiatrica Scandinavica* 2003;108(5):392-393.
30. Mathers CD, Bernard C, Moesgaard Iburg K, Inoue M, Ma Fat D, Shibuya K, Tomijima N, Xu H. *Global Burden of Disease in 2002: Data Sources, Methods and Results*. Geneva: World Health Organization; 2003.
31. World Health Organization. *Maternal mortality in Viet Nam, 2000: an in-depth analysis of causes and determinants*. Manila: World Health Organization, Regional Office for the Western Pacific; 2005.

32. Kumar V. Poisoning deaths in married women. *Journal of Clinical Forensic Medicine* 2004;11:2-5.

33. Lal S, Satpathy S, Khanna P, Vashisht B, Punia M, Kumar S. Problem of mortality in women of reproductive age in rural area of Haryana. *Indian Journal of Maternal and Child Health* 1995;6(1):17-21.

34. Granja A, Zacarias E, Bergstrom S. Violent deaths: the hidden face of maternal mortality. *BJOG: an International Journal of Obstetrics and Gynaecology* 2002;109:5-8.

35. Murray L, Cooper P. Postnatal depression and infant development. *British Medical Journal* 1991;302(27 April):978-979.

36. Murray L. The impact of postnatal depression on infant development. *Journal of Child Psychology and Psychiatry* 1992;33(3):543-561.

37. Richter L. *The Importance of Caregiver-Child Interactions for the Survival and Healthy Development of Young Children*. Geneva: WHO Department of Child and Adolescent Health and Development, 2004.

38. Cooper P, Landman M, Tomlinson M, Molteno C, Swartz L, Murray L. Impact of a mother-infant intervention in an indigent peri-urban South African context. *British Journal of Psychiatry* 2002;180:76-81.

39. Patel V, Desouza N, Rodrigues M. Postnatal depression and infant growth and development in low income countries: A cohort study from Goa, India. *Archives of Disease in Childhood*, 2003;88(1):34-37.

40. Patel V, Rahman A, Jacob K, Hughes M. Effect of maternal mental health on infant growth in low income countries: new evidence from South Asia. *British Medical Journal* 2004;328:820-823.

41. Patel V, Prince M. Maternal psychological morbidity and low birth weight in India. *British Journal of Psychiatry* 2006;188(3):284-285.

42. Rahman A, Bunn J, Lovel H, Harrington R. Impact of maternal depression on infant nutritional status and illness: A cohort study. *Archives of General Psychiatry* 2004;61:946-952.

43. Ferri C, Mitsuhiro S, Barros M, Chalem E, Guinsberg R, Patel V, et al. The impact of maternal experience of violence and common mental disorders on neonatal outcomes: a survey of adolescent mothers in Sao Paulo, Brazil. *BMC Public Health* 2007;http://www.biomedcentral.com/1471-2458/7/209.

44. Walker S, Wachs T, Meeks Gardner J, Lozoff B, Wasserman G, Pollitt E, et al. Child development: risk factors for adverse outcomes in developing countries. *The Lancet* 2007;369:145-157.

45. Granthem-McGregor S, Cheung Y, Cueto S, Glewwe P, Richter L, Strupp B. Developmental potential in the first 5 years for children in developing countries. *The Lancet* 2007;369:60-70.

46. Patel V, Chisholm D, Kirkwood B, Mabey D. Prioritising health problems in women in developing countries: comparing the financial burden of reproductive tract infections, anaemia and depressive disorders in a community survey in India. *Tropical Medicine and International Health* 2007;12:130-139.

47. Barnett B, Parker G. Professional and non-professional intervention for highly anxious primiparous mothers. *British Journal of Psychiatry* 1985;146:287-93.

48. Armstrong K, Fraser J, Dadds M, Morris J. A randomized, controlled trial of nurse home visiting to vulnerable families with newborns. *Journal of Paediatric and Child Health* 1999;35:237-244.

49. Armstrong K, Fraser J, Dadds M, Morris J. Promoting secure attachment, maternal mood and child health in a vulnerable population: A randomized controlled trial. *Journal of Paediatric and Child Health* 2000;36:555-562.

50. Heh S, Fu Y. Effectiveness of informational support in reducing the severity of postnatal depression in Taiwan. *Journal of Advanced Nursing* 2003;42:30-36.

51. Wendland-Carro J, Piccinini C, Millar W. The role of an early intervention on enhancing quality of mother-infant interaction. *Child Development* 1999;70:713-721.

52. Eshel N, Daelmans B, Cabral de Mello M, Martines J. Responsive parenting: interventions and outcomes. *Bulletin of the World Health Organization* 2006;84(12):991-999.

53. Baker-Henningham H, Powell C, Walker S, Grantham-McGregor S. The effect of early stimulation on maternal depression: a cluster randomised controlled trial. *Archives of the Diseases of Childhood* 2005;90:1230-1234.

54. Chen C, Tseng Y, Chou F, Wang S. Effects of support group intervention in postnatally distressed women. A controlled study in Taiwan. *Journal of Psychosomatic Research* 2000;49:395-399.

55. Lara M, Navarro C, Rubi N, Mondragon L. Outcome results of two levels of intervention in low-income women with depressive symptoms. *American Journal of Orthopsychiatry* 2003;73:35-43.

56. Araya R, Rojas G, Fritsch R, Gaete J, Rojas M, Simon G. Treating depression in primary care in low-income women in Santiago, Chile: a randomised controlled trial. *The Lancet* 2003; 361:995-1000.

57. Patel V, Araya R, Chatterjee S, Chisholm D, Cohen A, De Silva M, Hosman C, et al. Treatment and prevention of mental disorders in low-income and middle-income countries. *The Lancet* 2007:44-58.

58. World Health Organization. World Health Report 2001. *Mental Health: New Understanding, New Hope*. Geneva: World Health Organization; 2001.

For further information contact:

Department of Reproductive Health and Research
World Health Organization
Avenue Appia 20, CH-1211 Geneva 27
Switzerland
Fax: +41 22 791 4171
email: reproductivehealth@who.int
www.who.int/reproductive-health

Department of Mental Health and Substance Abuse
World Health Organization
Avenue Appia 20, CH-1211 Geneva 27
Switzerland
Fax +41 22 791 4160
email: cabraldemellom@who.int
www.who.int/mental_health

United Nations Population Fund
220 East 42nd Street
New York, NY 10017
USA
Fax: +1 212 297 4915
email:izutsu@unfpa.org
www.unfpa.org
